# Improvement of Interoceptive Processes after an 8-Week Body Scan Intervention

**DOI:** 10.3389/fnhum.2017.00452

**Published:** 2017-09-12

**Authors:** Dana Fischer, Matthias Messner, Olga Pollatos

**Affiliations:** Department of Clinical and Health Psychology, Institute of Psychology and Education, Ulm University Ulm, Germany

**Keywords:** interoception, interoceptive accuracy, interoceptive sensibility, interoceptive processes, body scan, mindfulness

## Abstract

**Objective:** Interoceptive processes are defined as ability to detect sensations arising within the body. There is a growing body of research investigating ways of improving interoceptive processes. One promising approach increasing the attention to bodily sensations is the body scan (BS), a method stemming from mindfulness-based stress reduction. Research so far revealed only heterogenous findings of meditational practice and mindfulness-based stress reduction on interoceptive processes. Even more importantly, there is no study considering the effect of an 8-week BS intervention on interoceptive processes and the distinguishable subdomains of interoception. Therefore, the main objective of this research is to examine the effects of a BS intervention on different interoceptive subdomains over 8 weeks of training in two different samples.

**Methods:** In study 1, healthy participants executed a 20 min standardized audiotaped BS in the BS intervention group (*n* = 25) each day over 8 weeks. The control group (*n* = 24) listened to an audio book for the same amount of time. In study 2, the BS group (*n* = 18) was compared to an inactive control group (*n* = 18). In both studies, three measurement points were realized and interoceptive accuracy (IAc) – using a heartbeat perception task – as well as interoceptive sensibility (IS) – using confidence ratings for the heartbeat perception task and the subscale ‘interoceptive awareness’ of the Eating Disorder Inventory-2 (EDI-2) – were assessed.

**Results:** In study 1, we found, as a descriptive trend, IAc and confidence ratings to be increased irrespective of the condition. However, *post hoc* analysis revealed a significant improvement of IAc between T1 and T3 in the BS intervention only. IS revealed to be unaffected by the interventions. In study 2, we observed a significant positive effect of the BS intervention on IAc and confidence ratings compared to the inactive controls. As in study 1, IS (EDI-2) was unaffected by the intervention.

**Discussion:** The results highlight the fact that interoception can be improved by long-term interventions focusing on bodily signals. Further studies might focus on clinical samples showing deficits in interoceptive processes and could use other bodily systems for measurement (e.g., respiratory signals) as well methods manipulating body ownership.

## Introduction

Interoception is usually defined as sensing and processing of afferent signals from within the body and its visceral organs to the brain (Cameron, 2001; Craig, 2002). Such afferent signals include changes in heart rate, respiratory, or muscle contractions, which are typically classified into proprioception and visceroception (Vaitl, 1996). Proprioception describes the sensation of bodily signals from the skin and the musculoskeletal apparatus, whereas visceroception comprises signals arising from within the body or the inner organs (e.g., cardiovascular, respiratory, and gastrointestinal signals). The perception of these signals is related to bodily states such as thirst, hunger, desire, pain as well as the level of energy and stress (Vaitl, 1996; Herbert and Pollatos, 2008). However, the increasing interest in interoception is due to its close link to emotions, feelings, and the perception of disease-specific symptoms (Schandry et al., 1993; Tsakiris et al., 2011). Individuals with a more precise detection of internal bodily signals experience emotions more intensively, are better able to process such signals, respectively, and to regulate them (e.g., Craig, 2003; Barrett et al., 2004; Critchley et al., 2004; Herbert et al., 2011). In order to get a better understanding of the concept of interoception and the consequences of interindividual differences, it will be defined in more detail below.

Garfinkel and Critchley (2013) and Garfinkel et al. (2015) proposed a clear demarcation between the different nomenclatures and meanings of the term interoception. Recently, they developed a three-dimensional model of interoception and provided empirical support for the necessity to assess different aspects of interoceptive processes (Garfinkel et al., 2015). The model differentiates between interoceptive accuracy (IAc), interoceptive sensibility (IS), and interoceptive awareness. IAc has been proposed as the ability to detect signals from within the body and to objectively quantify the individual performance via behavioral testing. Typically, IAc is investigated using procedures related to cardiovascular perception. Most prominently, IAc is tested by the heartbeat detecting task (Schandry, 1981) or the heartbeat discrimination task (Whitehead et al., 1977; Katkin et al., 1983; Brener and Kluvitse, 1988). Another established test to investigate IAc is the water load test based on the sensation of gastrointestinal signals (Vaitl, 1996; Herbert et al., 2012). Actual methods include procedures to measure the sensitivity for respiratory signals (Garfinkel et al., 2016). However, the heartbeat detection task is widely used in research on interoceptive processes and represents the standard procedure to measure IAc (e.g., Eshkevari et al., 2014; Kindermann and Werner, 2014; Koch and Pollatos, 2014; Pollatos and Georgiou, 2016; Pollatos et al., 2016). Furthermore, Garfinkel et al. (2015) defined IS as the second dimension of interoceptive processes by the subjective belief and confidence in the internal bodily state, mostly assessed by self-report questionnaires (e.g., Body Perception Questionnaire; Porges, 1993). Another possibility to assess IS is to indicate one’s own subjective confidence in the performance of a specific IAc task (e.g., Ehlers et al., 1995; Khalsa et al., 2008; Parkin et al., 2014). Whereas the self-report questionnaires regarding bodily signals in general, the confidence rating is more specific because of its request directly after the IAc task. Otherwise, the confidence rating includes only one item, which is repeated assessed after every interval. In general, IS depends on cognitive processes such as body awareness and associated evaluations, memories as well as attitudes (Mehling et al., 2009). Lastly, the metacognitive or interoceptive awareness dimension combines objective and subjective measures. The aim is to analyze the accordance between confidence and accuracy. For analyses, Garfinkel et al. (2015) suggest receiver operating characteristics (ROC) curves or the trial-by-trial confidence-accuracy correlation.

To emphasize the importance of interoception, there is evidence of a disturbed IAc and/or IS in clinical and subclinical populations. For example, in patients with anorexia nervosa as well as overweight and obese people a reduced IAc and IS are observed (Fassino et al., 2004; Pollatos et al., 2008; Herbert and Pollatos, 2014; Fischer et al., 2016). On the other hand, patients with bulimia nervosa and healthy controls did not differ in IAc (Pollatos and Georgiou, 2016), but bulimic patients showed a reduced IS (Fassino et al., 2004; Pollatos and Georgiou, 2016). In addition, Eshkevari et al. (2014) found no differences in IAc between different patients with eating disorders and healthy controls, whereas IS was higher in patients with eating disorders than in the control group. Otherwise, people suffering from somatization disorder have a reduced IAc compared to healthy controls (Weiss et al., 2014). Similar results were found for patients with higher scores of alexithymia (Herbert et al., 2011; Pollatos and Gramann, 2012) and healthy participants with depression and anxiety symptoms as well as patients diagnosed with depression (Pollatos et al., 2009; Dunn et al., 2010; Terhaar et al., 2012). Moreover, findings concerning interoceptive abilities of patients with panic disorders are quite mixed. Ehlers et al. (1995) and Ehlers and Breuer (1996) demonstrated that patients with panic disorders (as well as generalized anxiety disorder) showed an enhanced ability to perceive cardiovascular signals. In contrast, subsequent studies on this topic found no reliable differences in interoceptive abilities in people suffering from panic or generalized anxiety (Barsky et al., 1994; Van der Does et al., 2000). According to Ehlers and Breuer (1996), those inconsistencies are due to different instructions and sample characteristics used in subsequent research (e.g., including patients with medication that might has affected their cardiovascular systems). Previous research proved this only for the accuracy of cardiovascular signals, but they found no evidence for other interoceptive abilities (Limmer et al., 2015). To conclude, there is evidence that interoceptive processes are disturbed in people with different mental problems. Because interoception has shown to be of importance for processes of emotion perception and regulation (e.g., Herbert et al., 2007; Wölk et al., 2014; Kever et al., 2015; Pollatos et al., 2015), it seems necessary to develop and study methods that possibly lead to changes in interoceptive processes.

Until now, it is not clear how to improve interoceptive processes most effectively. Recent studies proposed interoception – especially IAc – to be a trait-like sensitivity toward visceral signals (Herbert and Pollatos, 2012; Herbert et al., 2012). Thus, there is good reason to assume that interoceptive processes are positively affected by trainings of heartbeat or breathing sensitivity. Several empirical studies support the idea that heartbeat perception can be trained, especially through manipulation procedures such as food deprivation, physical effort, and changes of the autonomic cardiovascular system (Herbert et al., 2007, 2012; Herbert and Pollatos, 2012). Another opportunity are self-focused procedures with the increase of attention through a photo of oneself, self-relevant words, or the own mirror image (Tsakiris et al., 2011; Ainley et al., 2012; Pollatos et al., 2016). Mostly, these outcomes are only short-term effects and found in participants with a low IAc prior to the training. Whereas different self-focus procedures showed first evidence for improved interoceptive processes, previous research investigating techniques stemming from a Buddhism mindfulness tradition are rare and the evidence is mixed. Because mindfulness interventions are concerned with the idea of being present in the moment, focusing on the body and related signals in a non-judgmental way, such methods are of high interest in research on interoception.

One approach to train interoceptive processes might be the so called body scan (BS) as recently suggested (e.g., Parkin et al., 2014). The BS is – in addition to classical forms of meditation and mindful yoga – a central element of the mindfulness-based stress reduction program (MBSR) developed by Kabat-Zinn in 1979 (Kabat-Zinn, 1982; Kabat-Zinn and Hanh, 2009; Sauer-Zavala et al., 2013). Common to all three techniques is a mindfulness part, where attention is paid to different parts of the body (e.g., toes, back or head) as well as sensations (such as pain or muscle tension) in the present moment without any judgment or criticism of the moment to moment experience (Kabat-Zinn, 2013). Additionally, during the BS participants were also constantly instructed to focus on their breathing and heartbeat.

Empirically, many studies showed a positive influence of an 8-week MBSR training on different health related outcomes (e.g., stress reduction, cancer, depression, and heart disease) in people with and without psychiatric or somatic disorders (e.g., Astin, 1997; Davidson et al., 2003; Ramel et al., 2004; Eberth and Sedlmeier, 2012; Katterman et al., 2014; Ussher et al., 2014; la Cour and Petersen, 2015; Yang et al., 2016). To date, there is only limited evidence showing positive effects of the MBSR-program as well as the BS implemented separately on parameters of the cardiovascular system like heart rate, cardiac pre-ejection period, and cardiac respiratory sinus arrhythmia (Ditto et al., 2006; Zeidan et al., 2010). However, there is a growing body of research showing positive effects of practicing the BS continuously on body perception, concentration, awareness of different disease-specific symptoms, and stress reduction (Ditto et al., 2006; Weiss et al., 2010; Dreeben et al., 2013). It is presented here that there are no studies available exploring the single effect of the BS over 8 weeks on interoceptive processes which is especially important for the research.

So far, previous studies considering the relationship between meditation, MBSR/Mindfulness-Based Cognitive Therapy (MBCT), or other mindfulness programs on interoceptive processes showed contradictory results. With the focus on meditation, one study examined the influence of a 10-day Vipassana meditation course and found an increase of IAc in meditation novices compared to an inactive-control group (Krygier et al., 2015). In addition, it has been demonstrated recently that a mindfulness training can improve IS [subscales attention regulation, self-regulation, body listening and trusting of the multidimensional assessment of interoceptive awareness (MAIA); Mehling et al., 2012] in depressed patients compared to both an active-control group and a healthy control group (Fissler et al., 2016). The results of a neurophysiological study support these findings (Farb et al., 2013). Farb et al. (2013) observed structural and functional changes in brain areas (anterior insula regions and dorsomedial prefrontal cortex) linked to interoception through meditation practice. Nevertheless, expertise in meditation did not seem to have an influence on IAc (Khalsa et al., 2008). Accordingly, heartbeat perception accuracy was unaffected by whether participants were experienced meditators (Buddhist or Kundalini; experience ranging from 12 till 29 years) or novices. Similar results were found in studies of Nielsen and Kaszniak (2006) as well as Melloni et al. (2013). In both studies, the level of experience in meditation was not associated with differences in IAc. However, meditation experts felt significantly more confident in their interoceptive performance and experienced the task as easier compared to non-experts. Lastly, Parkin et al. (2014) investigated the relationship between trait mindfulness and IAc as well as IS. In line with the aforementioned results of Khalsa et al. (2008), no relationship between mindfulness and heartbeat perception (IAc) was found, but participants with higher mindfulness scores were more confident regarding their heartbeat perception performance (IS; see also Kok and Singer, 2016). There is only one study investigating the influence of an 8-week MBSR- or MBCT on IAc and IS (Parkin et al., 2014). Results showed no improvement in heartbeat perception, but participants felt more confident in their performance. Similar effects were observed in patients suffering from chronic pain and depression after 8 weeks of MBCT on the subscales self-regulation and not distracting of the MAIA (de Jong et al., 2016).

In a recent study of Bornemann et al. (2015), IS could be shown to improve after a 3 months BS intervention and breathing meditation compared to a retest control group, which was recruited to investigate effects of repeated testing. Findings of another recent study failed to provide support for a positive effect of a BS training on IAc and IS (Parkin et al., 2014). In detail, the researchers compared a group participating in 15-min BS over 1 week with a control group who listened to a relaxing beach sound for the same amount of time, and a waiting-list control group.

To conclude, evidence is mixed concerning the idea that mindfulness practice strengthens the ability to perceive bodily signals. Results show no difference between intensive programs (e.g., meditation, MBSR or MBCT) and the isolated performance of the BS. Nevertheless, it has been argued in all described studies that meditation or other specific mindfulness tasks should improve IAc and IS as well (e.g., Khalsa et al., 2008; Parkin et al., 2014). In most studies, an improvement of IS through meditation was found, MBSR/MBCT and BS measured by questionnaire or estimation of confidence. One reason for the BS not being able to change IAc and IS in previous studies might be the training interval. In most cases, the BS was conducted only for 1 week or even shorter (e.g., Parkin et al., 2014), and information about standardization is often missing. Furthermore, Parkin et al. (2014) discussed the differences between IAc and IS. They attributed, that IS will strengthen cognitive processes for the body perception due to the training, especially evaluations, memories as well as attitudes, which is independent from the real perception. This assumption is in line with the differentiation between IAc and IS by Garfinkel et al. (2015) and should be considered.

In an attempt to clarify mixed effects of BS practice on interoceptive processes, we conducted two studies where participants trained the BS over a period of 8 weeks with daily practice sessions. Healthy students were trained in the standard BS suggested by Kabat-Zinn (2013) and longitudinal effects on interoceptive processes were assessed subsequently. This is the first study using three time points to measure outcomes of a BS training (T1: at the beginning; T2: after 4 or 5 weeks and T3: after 8 or 9 weeks). We expected IAc and IS to improve from a BS training compared to participants listening to an audio book (control group in study 1) or an inactive (control group in study 2).

## Study 1

### Materials and Methods

#### Participants

Both studies were approved by the Institutional Review Board of the Ulm University (Germany). A total sample of 50 healthy students aged 18–34 (79% female) were recruited by advertisements placed across the campus and an e-mail announcement. One participant did not complete all parts of the study resulting in data from 49 students available for the analyses. Participants were randomly assigned to the BS group (intervention group) or audio book group (control group), dependent variables were assessed at three time points.

The sample of the intervention group included 25 participants with a mean age of 22.4 (*SD* = 3.4), six of them were male (**Table 1**). Twenty-four participants, on the other hand, were assigned to the control condition, including four male students, with a mean age of 22.6 (*SD* = 4.2) both groups did not differ in age [*t*(47) = -0.208; *p* = n.s.; see **Table 1**]. Furthermore, participants should report their individual experiences in different relaxation and meditation techniques (i.e., BS, MBSR, Yoga or Pilates) ranging from regular experience (every week) over occasional to no experience at all. Six participants reported regular experience, six participants occasional and 13 participants no experience in the Body-Scan group, whereas in the control group three participants had regular experience, five occasional and sixteen no experience.^1^ Participants received monetary payment or course credit for their participation.

**Table 1:** Descriptive variables of body scan and audio book at T1.

	Body scan Mean (*SD*)	Audio book Mean (*SD*)	*t* (*df* = 47)	*P*
Age (years)	22.40 (3.4)	22.63 (4.2)	–0.21	0.836
BMI	22.12 (3.1)	22.10 (3.1)	0.03	0.981
IAc	0.60 (0.2)	0.56 (0.2)	0.90	0.371
IS (confidence)	4.36 (2.1)	3.96 (1.5)	0.77	0.448
IS (EDI-2)	2.21 (0.6)	2.06 (0.5)	0.88	0.385

*SD, standard deviation; BMI, body mass index; IAc, interoceptive accuracy; IS, interoceptive sensibility; EDI-2, Eating Disorder Inventory-2.*

#### Procedures and Materials

Participants were tested in the laboratory of the Clinical and Health Psychology Department in Ulm three times within an 8 weeks time period. When participants came to the laboratory for the first assessment, they were thoroughly informed about the study’s design and procedure, then they had to sign an informed consent to take part in the study. After a randomized assignment to one of the groups, participants provided demographics (e.g., age, gender, and education) and completed a battery of psychological questionnaires, including the State-Trait Anxiety Inventory (Laux et al., 1981) and the Beck Depression Inventory (Beck et al., 1996). Moreover, the interoceptive dimensions suggested by Garfinkel et al. (2015) were assessed as the main dependent variables: IAc (heartbeat tracking task) and IS [confidence and the subscale “Interoceptive awareness” of the German Eating Disorder Inventory-2 (EDI-2); Paul and Thiel, 2005]. The testing procedure was identical across all three time points (T1: at the beginning, T2: after 4 or 5 weeks and T3: after 8 or 9 weeks). Over 8 weeks then participants were instructed to perform the BS (concrete instructions are outlined below) each day for 20 min or to listen to an audio book for the same amount of time.

##### Interoceptive accuracy

For the measurement of IAc we used the heartbeat tracking task by Schandry (1981), where participants are instructed to take a comfortable and relaxed sitting position and to listen to their own heartbeats. However, participants had to count every heartbeat silently without checking the pulse manually or using any other technique for a better heartbeat perception (e.g., stop breathing). At the end of each interval participants had to report their counted heart beats. After a short training interval of 10 s, they completed four trials: 25, 35, 45 and 60 s. Importantly, they received no information about the length of these intervals. Participants simply got a start and stop signal from the research assistant. However, an electrocardiogram (ECG) assessed the objective heartbeats with non-polarizable Ag/AgCl electrodes placed at the right clavicular and the left chest. An amplifier system at a sampling rate of 1000 Hz was used. Finally, the heartbeat perception score (IA Score) was calculated using the individually counted and recorded heartbeats with the following formula:

$$\begin{array}{c} IAScore=\frac{1}{4}\sum \left(1-\frac{\left(\left|\mathrm{recorded}\mathrm{heartbeats}-\mathrm{counted}\mathrm{heartbeats}\right|\right)}{\mathrm{recorded}\mathrm{heartbeats}}\right) \end{array}$$

The IAc score can vary between 0 and 1, with higher scores indicating a better heartbeat perception and consequently a better IAc.

##### Interoceptive sensibility

To quantify IS, participants had to report their subjective confidence in the heartbeat perception performance. After each trial they were asked to indicate their confidence on a 10-point Scale ranging from “total guess/no heartbeat awareness” (=0) to “complete confidence/full perception of heartbeat” (=10). Beyond that, the subscale ‘interoceptive awareness’ from the German EDI-2 (Garner, 1984; Paul and Thiel, 2005) was used. The 10 items are self-rated on a 6-point Likert-scale from 1 (*never*) to 6 (*always*). Formed to an index, they represent the subjective ability to identify bodily signals, especially hunger or satiety. Higher scores indicate problems in IS.

##### Intervention: body scan vs. audio book

Participants randomly assigned to the BS group were handed out a smartphone with the app movisensXS, Karlsruhe (Germany). The aim of the app was to control the completion of the BS at every single day. Participants were reminded routinely every day at 7pm. Also valence (“How do you feel at the moment?”), arousal (e.g., calm vs. tense, nervous vs. relaxed, excited vs. bored), and easiness (“How well could you follow the BS?”) before and after the BS were assessed on a daily basis. In contrast, participants in the audio book group received a USB-Stick containing a data file with the audio book (“The Madman’s Tale” from Katzenbach, 2008). However, they had the opportunity to decide on their own by which time of the day they listened to the BS or audio book. Both groups got a written instruction. This instruction included the following important aspects:

– a quiet place should be chosen where they do not get disturbed easily,
– smartphones, television, radio, or any other possible distraction should be turned off or avoided,
– intervention time should serve as a time-out from the daily routine,
– eyes should be closed if possible,
– for listening they should either sit on a chair or lay on the floor,
– every thought or experience should not be judged or criticized.

Participants in the BS group trained mindfulness each day by using a 20-min audio tape recording of a guided BS meditation edited from a longer script of Kabat-Zinn (2013). The audio tape included a short introduction sequence followed by the guided BS. In the BS sequence, participants’ attention was guided slowly and mindfully through the entire body from the part of the feet to the hips and to different parts of the face (e.g., lips, mouth, eyes, and forehead). Finally, participants should focus their attention to the whole body followed by a wake up phase.

Participants in the control condition listened to an audio book [German version of “The Madman’s Tale” from Katzenbach (2008)]. The psychological thriller is about a middle-aged man. He has been compulsory hospitalized into a psychiatric institution 20 years ago, which is closed down – since then, he is medicated for the voices in his head. A reunion on the ground of the hospital awakens traumatic experiences related to an unsolved crime which was responsible for the demise of the institution and now comes back to the present. Using an audio book as a control condition is a common control condition in different studies (e.g., Cropley et al., 2007; Mirams et al., 2013). In addition, by using these conditions we wanted to control for daily effort so that differences between the conditions are not due to differences in activity time.

#### Data Analysis

Statistical Package for Social Science (SPSS; version 21) was used for all statistical analyses reported below. All descriptive statistics and group differences regarding demographic data and interoceptive processes (heartbeat perception, confidence and subscale of EDI-2) were analyzed using independent *t*-tests. To analyze training effects of the BS intervention, a repeated measurement ANOVA was conducted with the group representing the between-subjects factor and IAc as well as IS representing the within-subject factor. The effect size η^2^ is reported and *p*-values less than 0.05 were set as statistical significant for all analyses. Otherwise contrast and *post hoc* analyses were conducted.

### Results

#### Sample Description and Questionnaire Data at T1

All demographic and interoception data are summarized in **Table 1**. As expected, both groups did not differ in age, BMI, IAc and IS (confidence and EDI-2) at T1.

#### Changes in Interoceptive Accuracy

**FIGURE 1:**
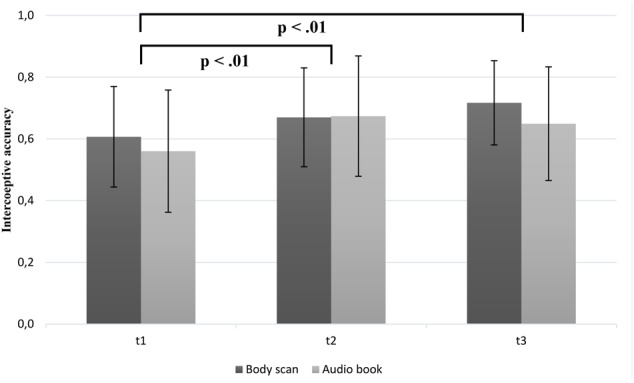
Mean interoceptive accuracy scores as a function of group (BS vs. audio book group) and time (note: significant group differences at all time points are not marked here).

With respect to IAc (**Figure 1**), the statistical analyses revealed a highly significant effect for time [*F*(2,94) = 11.17; *p* < 0.001; η^2^ = 0.19]. IAc increased in the BS group [mean T1 = 0.60 (*SD* = 0.16); mean T2 = 0.67 (*SD* = 0.16); mean T3 = 0.71 (*SD* = 0.14)] over time. *Post hoc* Bonferroni-adjusted analyses showed a significant increase only between T1 and T3 (*p* = 0.008). In contrast, the audio book led to an increase in IAc between T1 and T2 [mean T1 = 0.56 (*SD* = 0.20); mean T2 = 0.67 (*SD* = 0.19)], but a slightly decrease between T2 and T3 [mean T2 = 0.67 (*SD* = 0.19); mean T3 = 0.65 (*SD* = 0.18)]. However, *post hoc* analysis showed a significant increase between T1 and T2 (*p* = 0.006) and decrease between T1 and T3 (*p* = 0.04). Unexpectedly, the interaction between group × time [*F*(2,94) = 1.28; *p* = 0.28], as well as the main effect of group [*F*(1,47) = 0.77; *p* = 0.38] were not significant, indicating no differential effect of group membership on IAc. Regarding the contrast analysis and as expected, we found a linear trend for the BS group [*F*(24) = 11.20; *p* < 0.01], whereas there was a curvilinear trend [*F*(23) = 7.92; *p* < 0.01] and a linear trend [*F*(23) = 10.17; *p* < 0.01] for the audio book group.

#### Changes in Interoceptive Sensibility (Confidence)

**FIGURE 2:**
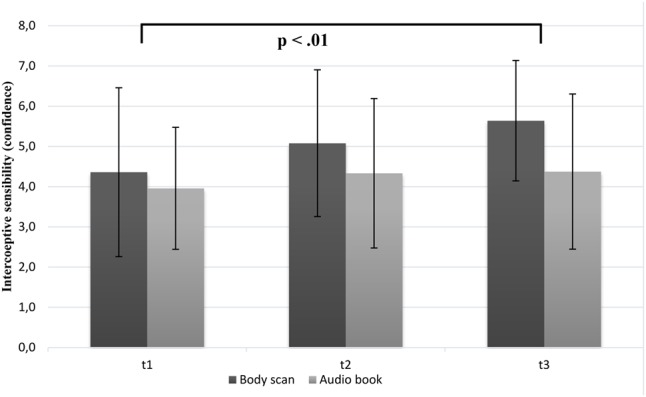
Mean confidence ratings (IS) as a function of group (BS vs. audio book group) and time (note: significant group differences at all time points are not marked here).

In line with our assumption, the results showed that interoceptive confidence differentiated significantly over time [*F*(2,94) = 6.89; *p* < 0.01; η^2^ = 0.12]. As it can be seen in **Figure 2**, the confidence rating increased more in the BS group from T1 to T3. Although, the main effect of group [*F*(1,47) = 3.35; *p* = 0.074] and the interaction between time and group [*F*(2,94) = 1.76; *p* = 0.178] failed to reach significance, separate *post hoc* analysis showed a significant increase regarding confidence only in the BS group from T1 to T3 (*p* < 0.01). Trend analyses showed significant linear trends for both groups [*F*_BS_(24) = 10.53; *p* < 0.01; *F*_AudioBook_(23) = 172.53; *p* < 0.01].

#### Changes in Interoceptive Sensibility (EDI-2)

Interoceptive sensibility was measured with the subscale “interoceptive awareness” of the EDI-2. Both groups differ only slightly regarding time. The IS of the BS group decrease from T1 (*M* = 2.21; *SD* = 0.65) to T3 (*M* = 2.07; *SD* = 0.58) and the mean score of IS for the control group is stable from T1 (*M* = 2.06; *SD* = 0.49) to T3 (*M* = 2.03; *SD* = 0.58). Accordingly, the main effect of time was not significant [*F*(2,94) = 1.189; *p* = 0.31]. Analysis of main effect regarding group did not contribute significantly to an effect [*F*(1,47) = 0.361; *p* = 0.551]. Finally, the repeated measurements ANOVA for the group BS versus audio group revealed no significant group × time interaction [*F*(2,94) = 0.423; *p* = 0.656].

### Discussion

Supporting our assumption, we found significant linear trends for IAc and IS (confidence). *Post hoc* analyses revealed significant differences for the IAc between T1–T2 and T1–T3 as well as for the confidence between T1 and T3. Contrary to our hypothesis, we found no alteration regarding IS assessed by questionnaire. There are promising results in the BS group, where we need to tie up and consider some limitations of study 1 to avoid similar results in the control group. Firstly, it could be possible, that the audio book condition has a mindfulness-effect, too. For both groups we used a similar instruction with comparable information (i.e., a place without any disturbance, time-out from daily routine, do not judge or criticize). Thus, participants should focus on their self and use this time for themselves in both task. This leads to a comparable mindfulness setting, especially because of the perception of the present moment. Moreover, the audio book control condition could be influenced by their motivation. Participants described the audio book as exciting and reported that they are highly motivated to hear the next chapter the next day. Compared to the audio book, the BS audio file was the same the entire time. Thus, we can speculate that the groups are different in their motivation level. Nonetheless, it seems that the BS has beneficial effects on interoceptive processes (see *post hoc* and contrast analyses). Having these points and limitations in mind, we decided to start a study 2 with an inactive control group, indicating a group with no intervention over the same time.

## Study 2

### Materials and Methods

#### Participants

Thirty-six participants took part in this study. Study 2 was identical to the experimental design of study 1 where participants’ interoceptive abilities were tested at three time points after 8 weeks of individual training of the BS group compared to an inactive control group. Participants were randomly assigned to one of the groups.

Eighteen female participants (*M*_Age_ = 22.2; *SD* = 2.8) of the intervention group were compared to 18 participants of the inactive control condition (*M*_Age_ = 22.8; *SD* = 3.3; see **Table 2**). There was no difference in age between groups [*t*(34) = -0.594; *p* = n.s.]. Regarding meditation experience, participants’ experience in the BS group ranging between (1) regular experience (*n* = 3), (2) experience with once in a while (*n* = 7) and (3) no experience (*n* = 8). In contrast, one participant of the inactive control group had regular experience, nine participants indicated experience with once in a while and eight participants reported no experience. At the end of the three laboratory sessions participants received course credit or were compensated monetarily.

**Table 2:** Descriptive variables of the body scan- and inactive control condition at T1.

	Body scan Mean (*SD*)	No-intervention Mean (*SD*)	*t* (*df* = 47)	*P*
Age (years)	22.17 (2.8)	22.78 (3.3)	–0.59	0.556
BMI	21.87 (2.2)	22.39 (3.3)	–0.57	0.575
IAc	0.53 (0.2)	0.63 (0.2)	–1.64	0.110
IS (confidence)	4.31 (1.7)	3.79 (2.0)	0.83	0.412
IS (EDI-2)	2.17 (0.6)	2.29 (1.2)	–0.42	0.680

*SD, standard deviation; BMI, body mass index; IS, interoceptive sensibility; IAc, interoceptive accuracy; EDI-2, Eating Disorder Inventory-2.*

#### Procedures and Materials

Parallel to the procedure of study 1, all participants were initially informed about the study, then instructed about the procedure and asked to sign a written informed consent. Also in line with study 1, demographic variables and IAc and IS were assessed as outcome variables. Participants in the BS group trained themselves in a body-scan meditation over 8 weeks, 20 min each day (for a detailed description see study 1), whereas participants in the inactive control group came three times to testing sessions in the laboratory of the Clinical and Health psychology Department in Ulm without performing a specific task in the meantime.

#### Data Analysis

We used the same data analysis procedures as in study 1.

### Results

#### Sample Description and Questionnaire Data at T1

**Table 2** summarizes all demographic and interoception data. Both groups did not differ in any of these variables.

#### Changes in Interoceptive Accuracy

**FIGURE 3:**
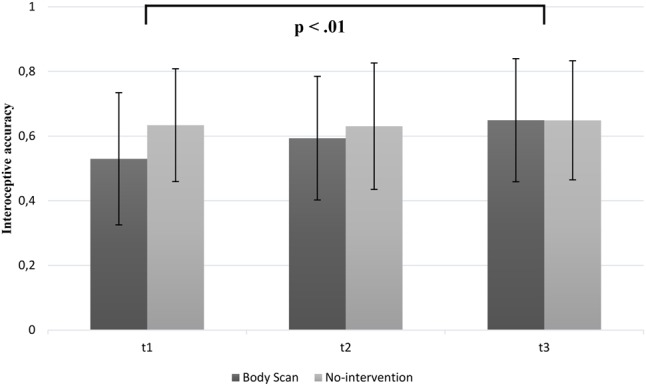
Mean interoceptive accuracy scores as a function of group (BS vs. inactive group) and time (note: significant group differences at all time points are not marked here).

**Figure 3** summarizes the changes in IAc over 8 weeks. Mean scores of the BS group regarding the heartbeat perception showed an increase from T1 to T3 [mean T1 = 0.53 (*SD* = 0.20); mean T2 = 0.59 (*SD* = 0.19); mean T3 = 0.65 (*SD* = 0.19)], whereas the inactive group remained stable in IAc [mean T1 = 0.63 (*SD* = 0.17); mean T2 = 0.63 (*SD* = 20); mean T3 = 0.65 (*SD* = 0.18)]. Consequently, there was a significant main effect for time [*F*(2,68) = 5.33; *p* < 0.01; η^2^ = 0.12], indicating an increase of IAc over 8 weeks. *Post hoc* analysis revealed a significant increase between T1 and T3 for the BS only (*p* < 0.05). As expected, we found no significant increase in IAc over time for the inactive control group with regard to the *post hoc* Bonferroni-adjusted analysis. Furthermore, we found a significant group × time interaction [*F*(2,68) = 3.28; *p* < 0.05; η^2^ = 0.08]. No significant main effect of the group [*F*(1,34) = 0.64; *p* = 0.43] was observed. However, as it can be seen in the mean IAc scores at T1, there is a difference of 0.10 between the groups in favor of the control group. This difference is statistically not reliable [*t*(34) = -1.64; *p* = 0.11]. Moreover, the results were supported by the conducted contrast analyses, showing a significant linear trend for the BS group [*F*(17) = 7.09; *p* < 0.01] and no significant trend for the inactive control group [*F*(17) = 0.49; *p* = 0.49].

#### Change in Interoceptive Sensibility (Confidence)

**FIGURE 4:**
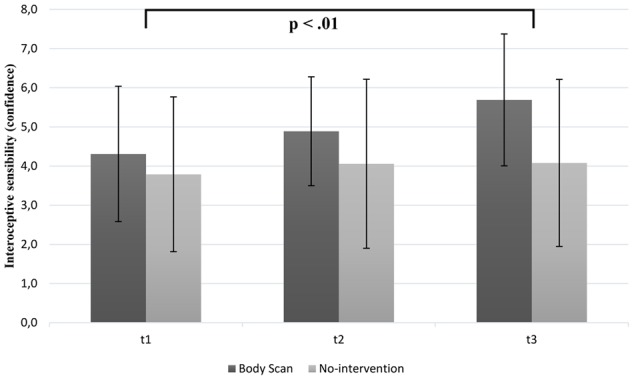
Mean confidence ratings as a function of group (BS vs. inactive group) and time (note: significant group differences at all time points are not marked here).

Mean values for the confidence ratings of the heartbeat perception score are depicted in **Figure 4**. These ratings were significantly higher at T3 than at T1 in the BS group [mean T1 = 4.31 (*SD* = 1.73); mean T3 = 5.69 (*SD* = 1.68)] compared to the inactive control group [mean T1 = 3.79 (*SD* = 1.98); mean T3 = 4.08 (*SD* = 2.13)]. We observed a significant group × time interaction [*F*(2,68) = 3.11; *p* = 0.05; η^2^ = 0.07] and a significant main effect of time [*F*(2,68) = 6.89; *p* < 0.01; η^2^ = 0.16]. Additionally, no main effect of group [*F*(1,34) = 3.06; *p* = 0.09] was found. *Post hoc* analyses revealed a significant improvement of the rating between T1 and T3 (*p* < 0.05) as well as T2 and T3 (*p* < 0.01) in the BS group. As assumed, *post hoc* analyses showed no significant differences for the inactive control group. Similar to the contrast analyses revealed for the IAc, we found a significant linear trend for the BS group [*F*(17) = 8.43; *p* < 0.01] and no significant trend for the inactive control group [*F*(17) = 2.46; *p* = 0.14].

#### Change in Interoceptive Sensibility (EDI-2)

Not in line with our assumption, there was no increase of IS regarding the mean values of the EDI-2. Accordingly, no significant group-by-time interaction [*F*(2,68) = 0.70; *p* = 0.50] and main effect of time [*F*(2,68) = 2.56; *p* = 0.09] was revealed. Furthermore, no main effect of group [*F*(1,34) = 0.46; *p* = 0.50] was found.

### Discussion

Study 2 showed that an 8-week BS intervention significantly improved IAc – assessed by the heartbeat perception – and confidence during this heartbeat perception task (IS), but did not decrease problems of IS assessed by the EDI-2. These results were highlighted by the comparison with the inactive control group who did not show a change in IAc or IS. Therefore, it can be concluded, that the BS as a mindfulness task over 8 weeks has worked. Especially, the change between T1 and T3 regarding IAc and IS (confidence) was greater than in study 1. This could be an indication that the audio book from study 1 has a mindfulness effect, too, or other variables (e.g., instruction) were responsible for similar effects in the BS and audio book group. However, comparable with the first study, we used a small sample (*n* = 18). Additionally, we only collected data from a female student population, because of no registrations of male participants. It should be mentioned that there are differences regarding the IAc in the BS- and inactive control group to T1, which is not significant. Whereas the BS group started with 0.53, the control group had an IAc score of 0.63. In fact, both studies found that a BS over 8 weeks provided a positive influence on interoceptive processes (heartbeat perception and confidence).

## General Discussion

The purpose of the study was to examine the effects of a 20-min daily practiced BS on interoceptive processes. Congruent with other studies, showing mixed results regarding different interoceptive processes, we expected a higher improvement of these processes after an 8-week BS mindfulness training. Because of the mixed results in previous studies (e.g., Parkin et al., 2014; Bornemann et al., 2015; de Jong et al., 2016) and the use of only short trainings, we hypothesized that an 8-week practiced BS will show effects. This change of interoceptive processes is important because of the disturbed interoceptive processes in clinical and subclinical populations. Therefore, we conducted two studies in order to investigate effects of an insulated BS meditation on the different interoceptive processes – IAc and IS. The effects of a Body-Scan training were tested against two different control groups. In study 1, we used an active control group (audio book). Due to the null training results in study 1, we speculated that the audio book group, especially because of the similar instruction, has a mindfulness effect, too. To prevent this, we started study 2 with an inactive control group. We examined all groups three times over a period of 8 weeks. To our knowledge, this is the first study investigating effects of an 8-week BS intervention based solely on the BS on interoceptive processes.

### Body Scan and Interoceptive Accuracy

In both studies, we measured the IAc by the heartbeat perception task and the effect of these during an 8-week BS intervention. Both studies showed that participants were better able to detect their heartbeat more accurately when they trained themselves in scanning their body mindfully each day over the intervention period. Moreover, it should be noted that there are differences regarding the accuracy score in study 1 and 2. In contrast, the BS group in study 1 showed a higher score (T1 = 0.60) compared to study 2 (T1 = 0.53) at the beginning. In both studies, we used the same instruction and healthy students. Furthermore, the participants did neither differentiate in their daily practices of the BS nor in their mindfulness experiences. Nevertheless, we found initial support for the idea that paying attention to the body in a mindful way has a beneficial impact on interoceptive processes as it has been discussed elsewhere (Parkin et al., 2014). Unexpectedly, we observed a similar improvement of IAc for the audio book group, at least for the difference between T1 and T2 as well as T1 and T3 in study 1, too. Otherwise, we found a linear trend for the BS group as well as a linear and curvilinear trend for the audio book group. Consequently, it was still unclear whether the improvement was due to the BS intervention or due to other systematic influences in the intervention period. One critical point could be the same instruction for both groups (i.e., a place without any disturbance, time-out from daily routine, do not judge or criticize) and the relation to a mindfulness setting. Anyway, to date there are other mixed results available concerning the effects of mindfulness trainings on interoception (Parkin et al., 2014; Krygier et al., 2015) or comparisons between experienced and novice meditators in this regard (Nielsen and Kaszniak, 2006; Khalsa et al., 2008; Melloni et al., 2013). It should be noted that in the study of Parkin et al. (2014) only a short-term BS intervention of 1 week was used. With the point of the mindfulness effect during the audio book condition and the null intervention results of the short-term BS intervention in mind, we decided to start another study.

Our results of the second study were more in line with our assumptions. Firstly, the results for the IAc revealed that only the BS group improved the heartbeat perception performance over the 8 weeks and indicating a linear trend over all three measurements. The current findings are consistent with the assumptions of most researchers in this field, that meditation or BS practice can improve heartbeat perception score because of the higher focus on bodily signals (e.g., Parkin et al., 2014; Bornemann et al., 2015). Nevertheless, in most of these studies, the different intervention did not have an effect of the performance to perceive the heartbeat. To date, there is only the study of Parkin et al. (2014) investigating the effects of a BS intervention on IAc and the results are contrary to our findings. They found that participants did not improve their heartbeat perception skills during a 1-week BS intervention. One possible explanation for the different results, which were describe before, could be the length of the BS. Whereas the participants of Parkin et al. (2014) performed the BS for only 1 week, our participants conducted the BS for 8 weeks. This explanation can be supported by the *post hoc* analyses which revealed significant differences between T2 and T3 as well as T1 and T3. Thereby, we found no significant improvement between the first 4 weeks (T1 – T2). Similar to our study, Parkin et al. (2014) used three conditions: a BS as the intervention and sound as well as inactivity as control conditions. Otherwise, it should be noted that we firstly used the BS – audio book condition and 1 year later the BS – inactive condition in study 2. As pronounced earlier and a reason for the provision of study 2, the audio book condition might be responsible for a higher motivation of the participants as well as the instruction that participants should use the period as a time-out from the daily routine. Thus, the different instructions and motivation levels might explain the differences of the results between study 1 and 2.

### Body Scan and Interoceptive Sensibility via Confidence

Next, we investigated the confidence of the heartbeat perception task by the question how confident participant feel in their heartbeat perception after every interval. Garfinkel et al. (2015) described this as a good opportunity to combine the assessment of IAc and IS and to emphasize the relationship between the subjective (perceived) and objective (actual) variables of interoceptive processes. In this context, we observed that participants in the intervention group felt more confident in the heartbeat perception task after 8 weeks of BS training. Although the BS- and the audio book group of study 1 showed an improvement in the confidence both, *post hoc* analysis revealed only a significant improvement of the BS group between T1 and T3. Moreover, the confidence ratings of the audio book between T2 and T3 were relatively stable. Unfortunately and not in line with our assumption, both groups showed a linear trend. In study 2, the evidence was more meaningful and consequently we found only a higher confidence in the BS group. These results were confirmed by the *post hoc* analyses which revealed significant differences between T2 and T3 as well as T1 and T3 in the BS group. Additionally, we showed only a significant trend for the BS group. Interestingly, this higher confidence is concordant with the improved performance in the heartbeat perception task. The results are in line with recent findings from Parkin et al. (2014) who found higher confidence in interoception after an 8-week MBSR or MBCT training. However, they could not find an increase of the confidence after a 1-week BS intervention. This might give support for the idea that in order to improve interoceptive processes and confidence via mindfulness interventions we need time to cultivate or train over a longer period. Furthermore, the improvements between T2 and T3 as well as T1 and T3 in study 2 regarding the effects of an 8-week BS were supported more specifically here. This idea is also in line with findings of Nielsen and Kaszniak (2006) as well as Melloni et al. (2013), who found experts to feel more confident in their heartbeat perception performance than non-experts.

### Body Scan and Interoceptive Sensibility via Questionnaire

Finally, we observed no alteration for the IS (EDI-2) in both studies, independent of the group. The present findings are not in line with previous results in healthy and depressed participants investigating effects of mindfulness trainings and BS (Bornemann et al., 2015; de Jong et al., 2016; Fissler et al., 2016; Kok and Singer, 2016). Contrary results may be attributed to the different measurements of IS by questionnaires or only one question via smartphone. Garfinkel et al. (2015) suggested to use the Body Perception Questionnaire (Porges, 1993), but there are some more questionnaires like the MAIA (Bornemann et al., 2015). Former studies investigating IS and mindfulness trainings mostly used the MAIA (Bornemann et al., 2015; de Jong et al., 2016; Fissler et al., 2016). Kok and Singer (2016) measured IS through smartphone only by one question concerning how aware of their body participants felt. Because of these contradictory results, future research should involve the MAIA or the suggested BPQ. The EDI-2 focused on perception of hunger as well as satiety and maybe did not request different bodily signals like the BPQ does.

### Limitations and Future Research

Although the findings of the present research are overall very encouraging, there are some points that need to be discussed in more detail. First, participants in the BS intervention group received a smartphone to listen to the guided BS in both studies. Thereby we wanted to give a standardized audiotaped BS and control the performance of the participants. Nevertheless and despite this review, there is no guarantee that participants actually performed the BS every day. To monitor the performance of participants, the only possibility would be training sessions in the group or a teaching session. However, this is associated with a very high effort for researchers as well as for participants. In both studies, participants were able to train the BS easily at home at any time of the day. Many participants clearly communicated this as an advantage. Otherwise, a teaching session could be coupled with other disadvantages (e.g., deflection, strong pressure to adapt). Compared with MBSR programs, it is also usual to perform most of the exercises autonomously at home. Another limitation refers to the fact that our sample mainly consisted of female students. Especially in study 2, we only assessed female participants because we had no registration of male participants. At the same time, we did not assume any sex differences but future research might want to balance between male and female participants, persons with varying educational levels, or people varying in age and occupational status. As already mentioned, the BS focused on different parts of the body and not especially merely on heartbeat. Moreover, the only targeted bodily internal signals are cardiovascular and respiratory signals, but with a higher focus on breathing. Obviously, future studies should include different measurements of interoception. For example, Garfinkel et al. (2016) compared the heartbeat discrimination task (Whitehead et al., 1977; Katkin et al., 1983) with a respiratory resistance threshold task (Harver et al., 1993). The authors found no relationship between both tasks and different directions of correlations regarding cardiac interoception and tactile exteroception as well as respiratory interoception and exteroception. Furthermore, Durlik et al. (2014) emphasized in their study the interaction between interoception and exteroception in modulating self-consciousness. Consequently and because both constructs represent body awareness, it would be interesting to measure both in future studies. First results of Mirams et al. (2013) showed a positive effect of exteroception measured by tactile perception (using the Somatic Signal Detection task) after participants conducted the BS for 1 week.

## Conclusion

Our data provide consistent evidence for the influence of a BS intervention over 8 weeks on interoceptive processes. There are heterogeneous findings and statements about interoception as a trait or state variable (e.g., Schandry and Weitkunat, 1990; Ainley et al., 2012, 2013; Herbert et al., 2012). However there is first evidence, that especially participants with a low interoception can benefit from such a self-focus training (Ainley et al., 2012). Patients with different mental disorders showed low interoception (e.g., Pollatos et al., 2008, 2009; Weiss et al., 2014). Consequently, more research in clinical settings is needed to precisely clarify interoceptive processes in such samples and to find interventions for the improvement of these processes.

## Ethics Statement

Both studies were carried out in accordance with the recommendation of the Guideline by the Institutional Review Board of the Ulm University (Germany) with written informed consent from all subjects. All subjects gave written informed consent in accordance with the Declaration of Helsinki. The protocol was approved by the Institutional Review Board of the Ulm University (Germany).

## Author Contributions

DF, MM, and OP, designed the study, analyzed the data and interpreted them. DF collected data and wrote the first draft of the manuscript. DF, MM, and OP, have approved the final manuscript and critically discussed them.

## Conflict of Interest Statement

The authors declare that the research was conducted in the absence of any commercial or financial relationships that could be construed as a potential conflict of interest.

## Footnotes

1 We excluded no participants because of prior meditation experience.

## Abbreviations

BS: body scan
IAc: interoceptive accuracy
IS: interoceptive sensibility
